# Implementing Quality Improvement Initiatives to Improve the Use of Adolescent- and Youth-Friendly Health Services in Zou, Benin

**DOI:** 10.9745/GHSP-D-22-00223

**Published:** 2024-05-21

**Authors:** Josephat Avocè, Mamadou Kandji, Vanessa Mitchell, Koami Maurice Mensah, Hugues Gnahoui, Hawa Talla, René Jean Firmin Nakoulma, Cheikh Ibrahima Diop, Moussa Faye, Fatimata Sow, Krishna Bose

**Affiliations:** a The Challenge Initiative, Francophone West Africa Hub, IntraHealth International, Dakar, Sénégal.; b William H. Gates Sr. Institute for Population and Reproductive Health, Bloomberg School of Public Health, Baltimore, MD, USA.; c Organisation pour le Service et la Vie, Cotonou, Bénin.; d Clinton Health Access Initiative, Dakar, Sénégal.

## Abstract

**Introduction:**

In Benin, the unmet need for family planning services is especially high for adolescent girls and youth aged 15–24 years. The Challenge Initiative (TCI) supported the health system to assess and improve the quality of adolescent and youth sexual reproductive health services and enhance contraceptive uptake in 65 service delivery points (SDPs) of the Zou department.

**Program Description:**

Between June 2019 and March 2021, TCI supported the health districts in Zou to train an assessment team to complete 3 cycles of quality assessments (QAs) using a QA checklist adapted to the local context. Based on assessment scores, the SDPs were categorized into poor, moderate, or good to excellent quality. The SDP managers developed remedial action plans after each cycle and for each SDP and followed up with supportive supervision.

**Results:**

The first QA cycle showed that 52% of assessed SDPs achieved a good to excellent classification; by the second QA cycle, this reached 74%. However, the quality of adolescent- and youth-friendly health services regressed during the third QA cycle (during COVID-19 pandemic disruptions), when only 40% of SDPs achieved the good to excellent category. Between the first and second QA cycles, contraceptive uptake for adolescents and youth improved in the SDPs that had good or excellent quality of services, compared to the ones that were of lower quality (established significance level of 5% with a *P* value of .031).

**Conclusion:**

Further assessments could deepen our understanding of the internal and external factors that can affect service quality. The findings reinforce the importance of investing in quality improvement strategies to maximize the use of sexual and reproductive health services among adolescents and youth. They also underscore the need for a contextual and nuanced approach to ensure enduring results.

## Abstract (precis)

Implementing initiatives to improve the quality of adolescent- and youth-friendly health services resulted in improvements in quality assessment scores and increased contraceptive uptake among adolescents and youth.

## Key Findings

- To assess the quality of adolescent- and youth-friendly health services (AYFHS) at 65 service delivery points in Zou, Benin, a 15-item quality assessment (QA) checklist was implemented to measure adherence to AYFHS standards.
- Quality improvement initiatives and supportive supervision were implemented to address service delivery, contraceptive availability, and provider bias and proved to be effective mechanisms in boosting the quality of AYFHS after the first QA cycle and the use of contraceptive services.
- Due to the COVID-19 pandemic that occurred between the second and third QA cycles as well as internal factors, many service delivery points regressed in scores.

## Key Implications

- Local governments can adapt the QA tool and institutionalize it within their supportive supervision systems.
- National stakeholders should invest in quality improvement strategies to increase the potential use of sexual and reproductive health services among adolescents and youth.
- Program managers need to anticipate possible internal or external factors that could influence the quality of services and uptake of contraception and put in place plans to mitigate them.

## INTRODUCTION

The provision of high-quality sexual and reproductive health (SRH) services tailored to the needs of adolescents and young people is essential for overcoming the barriers that prevent this population’s use of health services.^1^ In Benin, low rates of modern contraceptive use among adolescents and youth can be attributed to multifaceted challenges, including issues related to method accessibility and the quality of health services.^2^ Evidence has shown that compared to adult women, adolescent girls often receive care and support that is suboptimal.^3^ Adolescents and youth cite a number of barriers that can affect their access to services, including a lack of awareness about where to obtain services; worries about stigma, embarrassment, confidentiality, and privacy; the cost of services; distance to service providers; need for parental consent; and negative provider attitudes.^4^^,^^5^ Thus, adolescents and youth use SRH services, including family planning (FP) services, much less frequently than adults do.^6^^–^^8^

Among adolescents aged 15–19 years in Benin, the data reveal notable variations in sexual activity patterns between females and males. In this age group, 47.9% of females reported having engaged in sexual activity at some point, compared to 29.1% of males. Moreover, a distinct gender gap is observed when it comes to early sexual initiation, as 12.0% of females acknowledged having had sex by age 15 years, compared to 6.2% for males in the same age range. Furthermore, these data highlight that, in the 15–19 years age group, a significant proportion of females (22.4%) remained sexually active in the last month, in contrast to 9.9% of males.^9^

In Benin, there is considerable unmet need for FP services for all women between 15–49 years of age, estimated at 32.3% for 2017–2018, which is even worse for adolescent girls aged 15–19 years at 33% and for those aged 20–24 years at 37%. Moreover, modern contraceptive prevalence nationally is 12.4% for women aged 15–49 years, with a notably lower figure of 5.2% among adolescent girls aged 15–19 years.^8^ Therefore, it is not surprising that, in Benin, approximately 50% of pregnancies occurring among adolescent girls aged 15–19 years are unintended, with 55% of these pregnancies leading to unsafe abortions.^3^ For pregnancies that are not terminated, young women face an elevated risk of complications and having low birth weight infants.^3^

In Benin, there is considerable unmet need for FP services for all women of reproductive age but particularly for adolescents and youth aged 15–24 years.

These data also reveal different pregnancy intentions and contraceptive needs among adolescent girls aged 15–19 years. If they were married or in a relationship, most of their recent pregnancies (76.7%) were wanted within 2 years, while others wanted to wait (22.5%), and very few did not want the pregnancy (0.9%). Among unmarried girls, fewer wanted pregnancies soon (39.8%), more preferred to wait (58.2%), and a small percentage did not want the pregnancy (2.0%). Many adolescent girls, especially those who are unmarried, have unmet needs for modern contraception (65.5% for unmarried girls and 33.0% for married girls).^9^ These data emphasize the importance of tailored FP services to address their varying needs and targeted efforts to improve the quality of and access to FP services, including demand generation, for adolescents and youth in need of services.^10^^,^^11^

Indeed, demand-generation activities are integrated within quality improvement (QI) and play a crucial role in reshaping the social norms surrounding the SRH of adolescents and youth. These activities challenge taboos, biases, and stigmas associated with these issues, fostering greater openness and better understanding of these subjects within society.^12^^,^^13^ Moreover, demand-generation activities promote open communication by facilitating honest and constructive discussions about SRH, both within families and in the community. This enables adolescents and young people to seek information, ask questions, and share their concerns. Concurrently, demand generation actively encourages young individuals to proactively seek SRH services, including contraception. Demand generation and quality of services reinforce each other, and both are needed to reduce barriers to AYSRH. Thus, these activities contribute to preventing unintended pregnancies and enhancing adolescents’ and youth’s access to high-quality SRH services by mitigating the social, cultural, and economic barriers that may hinder such access. Furthermore, as demand generation takes place around FP in communities, health facilities must be prepared to provide adolescents and youth with good quality services.^14^

The World Health Organization (WHO) emphasizes that to improve health outcomes and attain Sustainable Development Goals, particular attention must be given to reducing adolescent pregnancies and the risks of mortality and morbidity stemming from pregnancies.^3^ In accordance with this provision, Benin established a 2018–2022^15^ national multisectoral strategy for SRH of adolescents and youth to address these challenges and rectify gaps prevalent in the delivery of quality services within public and private service delivery points (SDPs).

This article addresses 2 guiding program questions aimed at enhancing the use of adolescent- and youth-friendly health services (AYFHS) in the Zou department of Benin through QI initiatives: (1) How can the quality of adolescent-responsive SRH services be enhanced? and (2) What is the impact of QI/quality assessment (QA) initiatives on AYFHS service quality? How might this, in turn, influence adolescent and youth contraceptive uptake?

## QUALITY IMPROVEMENT INITIATIVES FOR ADOLESCENT- AND YOUTH-FRIENDLY HEALTH SERVICES IN ZOU

**FIGURE 1:**
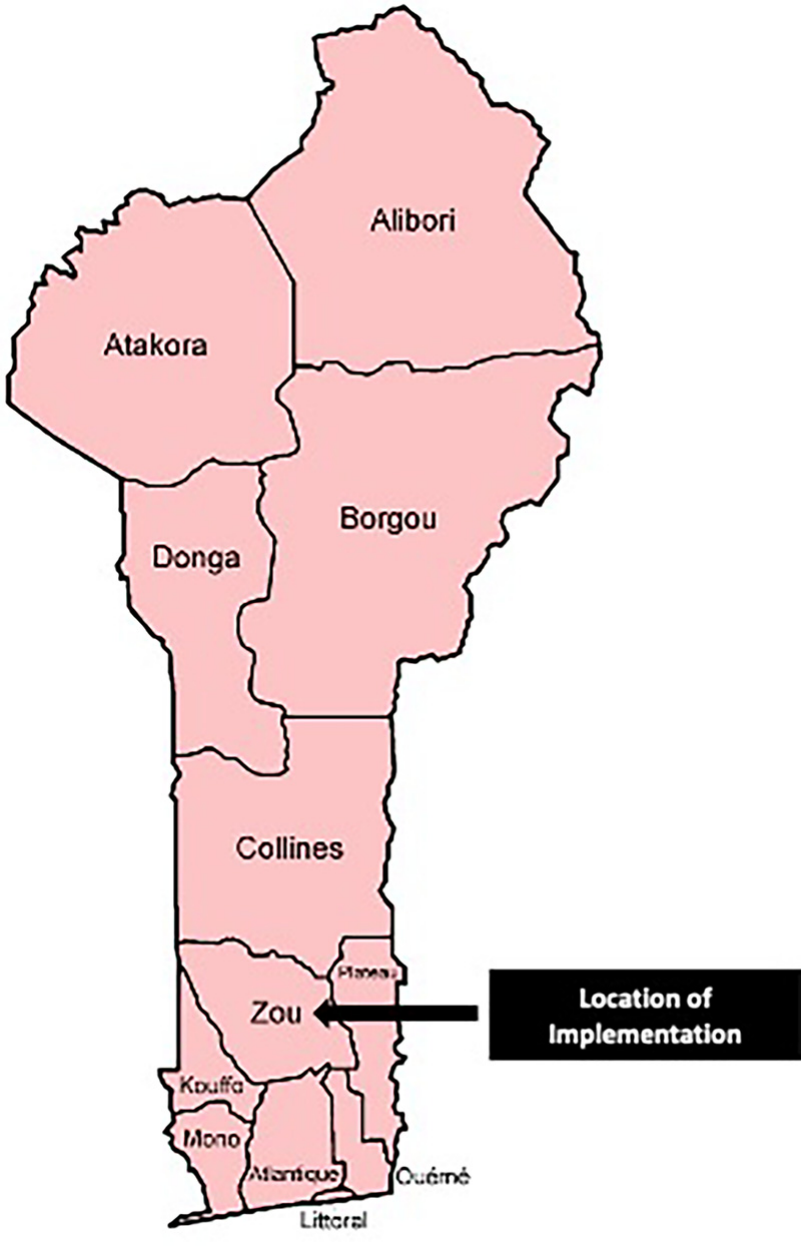
Map of Zou Department, Benin, Where Initiatives Were Implemented to Improve the Quality of Adolescent- and Youth-Friendly Health Services

**FIGURE 2:**
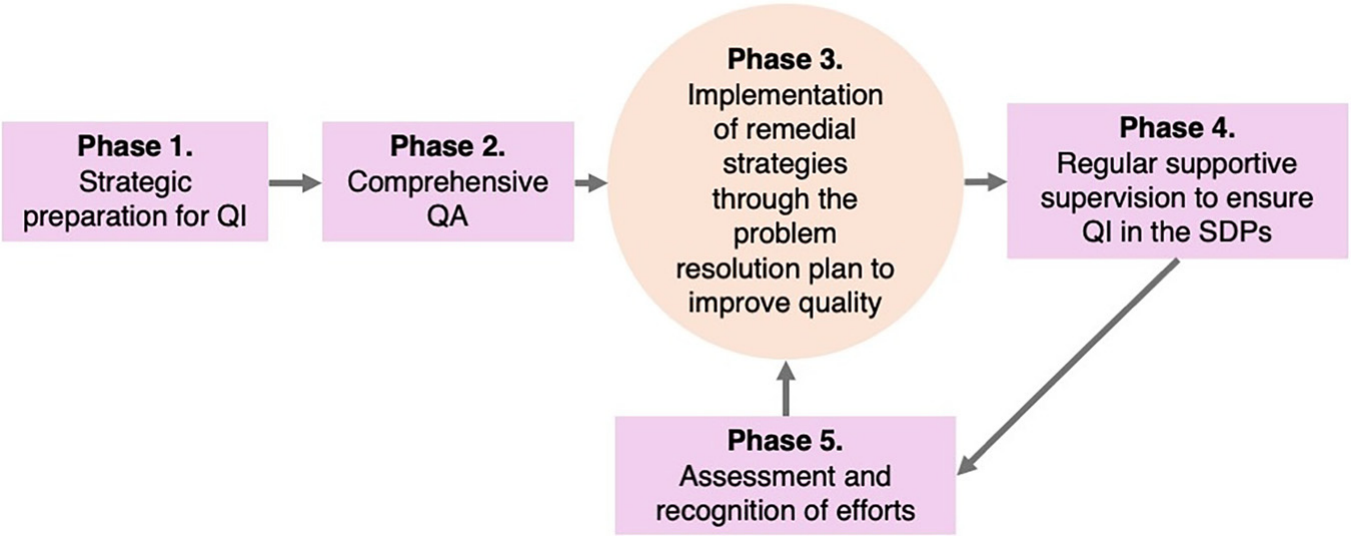
Quality Improvement Implementation Phases, Zou Benin Abbreviations: QA, quality assessment; QI, quality improvement; SDP, service delivery point.

In 2016, The Challenge Initiative (TCI), with funding from the Bill & Melinda Gates Foundation and supported by IntraHealth International, worked with local governments in Francophone West Africa to strengthen, scale up, and sustain high-impact FP practices that improve access to and uptake of contraceptive services.^16^ In June 2018, TCI secured funding to improve the quality of AYFHS and contraceptive accessibility for married and unmarried adolescents and youth ages 15–24 years, a subset of the broader demographic of women of reproductive age (WRA) 15–49 years.^17^ In the Zou department, one of 12 departments in Benin, the 9 communes collectively submitted the first expression of interest to TCI in 2018 for improving AYFHS in the SDPs (Figure 1) and thus were selected. A systematic QI approach encompassing 5 sequential phases (Figure 2) was implemented in Zou to improve adolescent and youth sexual and reproductive health (AYSRH) services, including contraceptive uptake.

### Phase 1. Strategic Preparation for Quality Improvement

This initial phase involved formulating clear objectives and anticipated outcomes, as well as delineating the pathway toward QI of services. SDPs were selected to ensure that service providers were in place. Before implementation began, Department Directorate of Health (DDS) authorities were oriented, their engagement with the process was ensured, and their official approvals were obtained. Thus, the QI process was developed and implemented in partnership between TCI and the DDS to ensure the process’ contextual relevance and methodological fidelity, including that of the Francophone West Africa QA Checklist, which was adapted from one initially developed and used by WHO’s Southeast Asia Regional Office. The resulting 15-item checklist was harmonized with WHO’s established Eight Global Standards for AYFHS^18^ (Table 1).

**TABLE 1.** Quality Assessment Framework^a^ Applied to Improve AYFHS Quality in Zou, Benin

**Criteria**	**Key Elements Addressed by the Assessment Criteria**
Provider capacity	1 Training and coaching of service providers on reducing bias (Global Standard 4: Providers’ competencies) 2 Attitudes of providers in counseling youth
Services and availability of commodities	3 Complete range of contraceptive products without stock shortages (Global Standard 3: Appropriate package of services) 4 Offers free FP/AYSRH services (Global Standard 3: Appropriate package of services) 5 Referral and counter-referral mechanism in place (Global Standard 3: Appropriate package of services) 6 Whole-site orientation on AYFHS conducted
Facility: equitable, respectful, nonjudgmental service characteristics, ensuring privacy and confidentiality	7 Tables of client rights in FP/AYSRH and charter of commitment of quality services visible (Global Standard 6: Equity) 8 Counseling room (respect for privacy, visual and auditory discretion) (Global Standard 5: Facility characteristics) 9 Secure record keeping (Global Standard 5: Facility characteristics)
Youth client feedback and engagement	10 Mechanism for collecting feedback from young clients (Global Standard 8: Adolescents’ participation) 11 Taking into account youth client perspectives and full participation of youth in the review and implementation of feedback to improve AYFHS (Global Standard 8: Adolescents’ participation)
Communication and demand generation with outreach to communities	12 Availability of communication support and materials (Global Standard 1: Adolescents’ health literacy) 13 Communication activities on FP/AYSRH within the service delivery points and with outreach to the community (Global Standard 2: Community support)
Data disaggregation by age, sex, and method of choice	14 Collection of SDPs’ attendance data disaggregated by sex and age (Global Standard 7: Data and QI) 15 Collection of contraceptive method use data by adolescents disaggregated by age, sex, and method (Global Standard 7: Data and QI)

Abbreviations: AYFHS, adolescent- and youth-friendly health services; AYSRH, adolescent and youth sexual and reproductive health; FP, family planning; QI, quality improvement.

^a^ Linkage to Eight Global Standards for AYFHS.^18^

To pretest the QA checklist, we selected both a public and a private SDP that offered a complete range of contraceptives and free FP services for adolescents and youth in the Collines department, which is outside the TCI intervention areas of the Zou. We chose Collines as a comparison site because it is similar to Zou in terms of its history (Collines used to be in the Zou department), sociocultural environment, and AYSRH data. These SDPs were chosen because they have a large volume of services, made efforts to improve providers’ attitudes toward adolescents and youth, have an efficient referral and feedback system, conduct awareness campaigns to stimulate demand for those who need services, and use specific tools for collecting data disaggregated by sex and age, all practices that align with WHO standards for youth-friendly services.

After the pretest, the checklist was finalized for application. To enhance the health system’s capacity to apply the finalized checklist, TCI supported the training of the QA team (Table 2). Additional key partners throughout the entire project were Zou adolescents and youth who belong to the Youth Transformational Leaders (YTL) association, supported by TCI. The YTL is a dynamic group of adolescents and youth aged 15–24 years, whether they are girls or boys, married or not, committed to promoting AYSRH. Organized as a nationally recognized movement with branches at the city, regional, departmental, and municipal levels, their mission is to transform the sociopolitical environment of AYSRH programs into a conducive environment, mobilize resources for AYSRH, and increase youth participation in decision-making and program implementation.

**TABLE 2.** Quality Assessment Team That Implemented the Checklist for All Three Cycles, Zou, Benin

	**No.**
Heads of FP/AYSRH services of the Departmental Directorate of Health	2
District and commune chief medical doctors	4
Midwife coordinators	3
Statistician, district, and region-level data manager	3
Nurse heads or midwives’ maternity managers	6
Youth Transformational Leaders Association representatives	6
The Challenge Initiative municipal focal points	2
Total	26

Abbreviations: AYSRH, adolescent and youth sexual and reproductive health; FP, family planning; QI, quality improvement.

Source: TCI Francophone West Africa project records.

The QA checklist was implemented with partners, including adolescents and youth, and stakeholders at multiple levels.

### Phase 2. Comprehensive Quality Assessment

First, SDP personnel were oriented on the importance of conducting QAs and on QA’s role in ongoing QI. Next, the assessment team observed and interviewed service providers about the quality of services for adolescents and youth in the SDPs. In addition, they assessed providers’ clinical knowledge, attitudes, and perceptions about the rights of adolescents and youth to access comprehensive contraceptive and reproductive health services. Using the QA checklist, the assessment team conducted a comprehensive assessment of the services, protocols, supplies, equipment, availability of informational materials, recordkeeping, referral systems, and maintenance of audio and visual confidentiality, among other features in all SDPs.

After the assessment, the team conducted a discussion with SDP staff about their strengths, areas for improvement, and pertinent insights gathered during the assessment process. The assessment team categorized the SDPs according to the performance scores established in the checklist. The scoring system was binary, with a score of “1” allocated when an assessment criterion was met and “0” allocated when it was not. Consequently, the maximum score attainable was 15, and the minimum was 0. The system comprised 3 categories—SDPs classified as poor received the lowest scores (0–9), indicating that critical areas related to AYFHS required immediate attention for improvement; moderate SDPs were facilities that needed some improvement for effective AYFHS service provision (10–11); and good or excellent SDPs had commendable performance related to AYFHS (12–15) (Table 3).

**TABLE 3.** Quality Assessment Scoring and Ranking of Service Delivery Points, Zou, Benin

**Score**	**Percentage**	**Quality Ranking**	**Honors**
0–9	0%–59%	Poor	No distinction
10–11	60%–79%	Moderate	No distinction
12–13	80%–89%	Good	Honors with 2 green stars
14–15	90%–100%	Excellent	Honors with 3 green stars

Source: The Challenge Initiative Francophone West Africa Quality Assessment Checklist.

To address identified deficiencies in AYFHS, TCI and the SDP management teams developed problem resolution plans using a systemic approach to tackle the barriers faced by adolescents and youth when seeking high-quality SRH services.^19^^–^^21^ Special emphasis was placed on service delivery and improving the skills of health personnel to provide quality services. Similarly, actions were implemented to improve the attitudes of SDP personnel to reduce prejudices and stigmatization toward adolescents and youth.^22^

### Phase 3. Implementation of Remedial Strategies

To transform poor-quality SDPs into good- or excellent-quality SDPs, QI actions were undertaken through the implementation of the problem resolution plans. Complementary interventions, strategically aligned with the overall mission of improving quality, were seamlessly integrated to enhance the impact on the delivery of FP services. Adolescents and youth in health facilities were informed about FP through education sessions, their FP needs were identified, and, depending on the situation and with their consent, were referred to the appropriate providers. In addition, to engage adolescents and youth on topics related to SRH, social and behavioral change communication activities were organized in school and workplace settings and reinforced by radio and digital campaigns through social media.

To address inherent biases, 87 of 88 providers received comprehensive AYSRH training focused on QI approaches. The DDS developed and conducted comprehensive whole-site orientations (WSOs) on AYFHS at 83 of 88 SDPs in Zou for all clinical and nonclinical staff, including ancillary staff such as cleaners and watchmen. WSOs aim to create SDP-wide support for AYFHS and to reduce discriminatory, judgmental, and disrespectful service delivery approaches toward youth seeking services.

TCI assisted the health system in improving data collection and data quality by developing and deploying 600 copies of registers in SDPs to collect disaggregated age and gender data and by organizing data control and validation workshops. To prevent shortages of contraceptive products, TCI trained coaches to assist managers of distribution depots and pharmacies in the health districts in better stock management, including for contraceptives.

Because adolescents and youth often encounter financial barriers when accessing SRH services, TCI collaborated with YTL, municipalities, and SDPs to establish vouchers to enable adolescents and youth to access services at no cost. Municipalities entered into agreements with service providers who submitted their invoices to municipalities for reimbursement. As a result, 1,449 free vouchers were distributed, and through YTL advocacy activities, 2 million CFA was mobilized to pay for the submitted invoices. In addition, free access to contraception services was provided by organizing days for the free provision of FP services.

A fundamental element of the QA and overall QI process was to ensure increased and consistent youth engagement, thereby fostering a sense of co-ownership within the youth cohort. TCI assisted the municipalities of Zou in establishing the YTL to actively engage adolescents and youth in the QI process, integrate their perspectives, collect their feedback on the quality of services, and implement corrective measures with their participation, ultimately enhancing their satisfaction.

A fundamental element of the QA and overall QI process was to ensure increased and consistent youth engagement, thereby fostering a sense of co-ownership within the youth cohort.

### Phase 4. Regular Supportive Supervision to Ensure Quality Improvement

In accordance with the Ministry of Health guidelines, supervision was conducted at the department, health district, and SDP levels. Twice a year, each department organized routine supervision of the SDPs lasting 3 days with the participation of 2 national representatives from the Directorate of Maternal and Child Health. Every quarter, the health district conducted supervision with 2 members of the department level. On a monthly basis, SDP managers conducted supervision at the SDPs with community health workers.^23^ Members of these supervision teams partnered with others to form the groups responsible for the QA/QI process, incorporating several aspects into the supervision tools and checking them during the sessions. These aspects include the reception and attitudes of providers, the availability of a full range of contraceptives and free FP services for adolescents and youth, referral and feedback systems, the organization of demand-generation activities, and the collection of data disaggregated by sex and age. However, resource-related constraints, such as the lack of qualified staff, time, equipment, and logistics, limited the regularity of these supervisions, which were often reduced to a single session per semester.^24^

### Phase 5. Reassessment and Recognition of Efforts

This phase encompassed the repeated implementation of QA (Phase 2), typically undertaken at intervals spanning 9 to 12 months after the previous QA. Additionally, commendable contributions were acknowledged through a reward mechanism, which recognized exemplary SDP personnel.

## RESULTS

As part of our study, we selected 65 health centers that participated in 3 cycles of service quality evaluation (June 2019, March 2020, and March 2021) and classified them according to their performance into 3 categories: poor, moderate, and good to excellent. We extracted health center data from DHIS2 from 2020 and 2021 on new and continuing acceptors, specifically for those aged 15–24 years, for both sexes. It is noteworthy that our analysis does not rely on the “contraceptive prevalence rate,” “utilization rate,” or “couple-years of protection” but rather on the cumulative volume of new and continuing acceptors, considered as an indicator of client volume, without any further adjustment.^25^ We used the T-test to compare the average usage between health centers in the poor and good categories, employing SPSS Version 21 and XLSTAT 2023 for statistical analysis. This approach allowed us to identify variations in the use of FP services and to formulate evidence-based recommendations to improve the quality of services offered to targeted populations.

### Quality Assessment and Improvement Trends in Zou: A Three-Cycle Analysis

This initial outcome addresses the first guiding program question on enhancing the quality of AYFHS. In June 2019, the first QA cycle showed that 52% (34/65) of assessed SDPs achieved a “good” classification (Table 4). The second QA cycle conducted in March 2020—after the rollout of the problem resolution plan and regular coaching and immediately preceding COVID-19-related disruptions—showed a substantive increase to 74% (48/65) of SDPs categorized as good. However, during the third QA cycle in March 2021, the quality of AYFHS showed a noteworthy regression, with only 40% (26/65) of SDPs achieving the good category, which is below the observed values for the first QA cycle.

**TABLE 4.** Overview of QA Outcomes From Three QA Cycles, Zou, Benin

			**SDP QA Outcomes**		
			**Poor**	**Moderate**	**Good**
**Cycle**	**Month, Year**	**No. of SDPs Evaluated**	**No. (%)**	**No. (%)**	**No. (%)**
First	June 2019	65	2 (3.1)	29 (44.6)	34 (52.3)
Second	March 2020	65	9 (13.8)	8 (12.3)	48 (73.9)
Third	March 2021	65	9 (13.8)	30 (46.2)	26 (40.0)

Abbreviations: QA, quality assessment; SDP, service delivery point.

Source: The Challenge Initiative Francophone West Africa project records.

### Enhanced Contraceptive Uptake Through Quality Improvement Initiatives

Related to our second guiding program question, documenting whether contraceptive uptake for adolescents and youth increased in response to QI efforts was an important aspect of our analysis. During the first QA cycle in June 2019, only 2 SDPs were identified as providing poor quality AYFHS, and data relating to the contraceptive uptake were not yet disaggregated by age in the Benin DHIS2. As a result, we excluded this assessment cycle from our analysis.

Our focus then shifted to the comparative analysis of contraceptive uptake among adolescents and youth aged 15–24 years across good-quality and poor-quality SDPs of the second and third assessment cycles of March 2020 and March 2021. Table 5 summarizes the client volume in 2020 and 2021 among female and male adolescents and youth aged 15–24 years by different SDP categories in Zou.

**TABLE 5.** Volume of Adolescents and Youth Clients Aged 15–24 Years by SDP QA Category, Zou, Benin

**Cycle**	**Year**	**No. of SDPs evaluated**	**Client Volume, No.**			
			**Poor**	**Moderate**	**Good**	**Total**
Second	2020	65	1,229	1,423	13,104	15,756
Third	2021	65	1,765	6,278	6,920	14,963

Abbreviations: QA, quality assessment; SDP, service delivery point.

Source: Benin DHIS2.

The moderate SDPs represented health centers in transition and served as indicators of the dynamics of service quality or its progress indicators. Their inclusion in the analysis could have introduced additional variability in the results. Aiming for clearer results, we chose not to include the moderate SDPs in the analysis of adolescent and youth contraceptive uptake. The analysis of 2020 data showed that contraceptive uptake among adolescents and youth was significantly different across good- and poor-quality SDPs. At the 5% significance level, the *P* value was .031 (Table 6). However, the comparison of contraceptive uptake among adolescents and youth aged 15–24 years in Zou in 2021 across good- and poor-quality SDPs did not reveal a statistically significant difference. At a significance level of 5%, the *P* value is .264.

**TABLE 6.** T-test for Comparison of Contraceptive Uptake Between Good-Quality and Poor-Quality Service Delivery Points, Zou, 2020 and 2021

Year	t (Observed Value)	Degrees of Freedom	Two-Sided *P* value	Mean Difference	Standard Deviation Difference	95% Confidence Interval Around the Difference of Means
2020	2.211	55	.031	136.4444	61.7182	12.7585, 260.1304
2021	0.136	33	.264	70.0427	61.6511	−55.3874, 195.4729

Source: Benin DHIS2.

## LESSONS LEARNED

Our findings suggest that QI of AYFHS is feasible and measurable using a simple QA checklist followed by supportive supervision and that QI can lead to increased use of contraceptive services.

As evidenced in the third round, QI is a dynamic process, and contraceptive use can regress if internal (e.g., age and gender disaggregation in DHIS2, supply chain system, demand generation, availability of trained providers) or external factors (e.g., COVID-19 pandemic) affect the health system.

QI is a dynamic process and contraceptive use can regress if internal or external factors affect the health system.

The post-assessment problem resolution plan and remedial action were essential to improving QA scores. Moreover, emerging data show that improved service quality is associated with increased contraceptive uptake, which would have tremendous implications in support of QI.^26^^,^^27^ The transformation we observed exemplified the potential effectiveness of focused QI approaches in creating an environment that attracts increased numbers of adolescents and youth clients for contraceptive uptake.

In the context of challenges associated with implementing SRH services tailored for adolescents and youth in resource-limited environments, integrating these services within existing health systems presents itself as a viable strategy to reduce costs and ensure sustainability. This approach not only optimizes the use of already available infrastructure and resources but also facilitates access for adolescents and youth people by reducing the stigma associated with seeking such services.^19^^,^^20^

By using the health system building blocks to develop improved quality initiatives, these integrated strategies directly contributed to the overall strengthening and sustainability of the health system while specifically addressing the needs of youth.^21^^,^^28^ Implementing targeted interventions, such as WSOs, actively managing contraceptive logistics to prevent stock-outs, reducing biases among service providers, involving adolescents and young people in QI and innovation processes, organizing awareness activities, and collecting disaggregated data by sex and age, is essential to meet the SRH needs of adolescent and youth.^29^ They offer practical and economical solutions that can be implemented without the need for heavy infrastructure investments.

## CHALLENGES AND LIMITATIONS

There are certain inherent challenges in implementation and limitations in this study.

First, the findings are specific to the Zou context and may not be readily generalizable to other contexts.

Having quality data and age- and gender-disaggregated data on health service utilization, including FP services, presents challenges in many countries, though in Benin, age disaggregation of routine data in SDPs began across the country and notably for FP since 2020. Another limitation of the QA/QI data is that they rely on interviews of SDP staff that can be biased to obtain more favorable results.

Although the initial improvements in service quality following the implementation of the QI process were promising, the subsequent decline in quality, particularly in certain SDPs, was attributed to internal factors, including stock-outs of supplies, provider attitudes, and lack of demand-generation activities. These factors were further exacerbated by the COVID-19 pandemic, causing more frequent stock-outs of essential supplies, reduced demand-generation activities, and decreased provider availability for service provision. These events underscore the dynamic nature of the QI process, as it can be influenced by various internal and external factors, including unexpected disruptions like a public health crisis.

## RECOMMENDATIONS

The observed regression in service quality emphasizes the importance of maintaining ongoing QI efforts to counteract the potential adverse effects of such factors. To ensure that health system conditions remain conducive to the delivery of high-quality services, it is crucial to address both internal factors (attitudes and providers training, availability of a full range of contraceptives and free FP services for adolescents and youth, referral and feedback systems, organization of demand-generation activities, and collection of data disaggregated by sex and age in DHIS2) and external elements (COVID-19 pandemic^6^ and restrictive laws and norms that make the sociopolitical and legal environment unfavorable^30^) that can influence service quality in SDPs. This may include strategies to enhance supply chain management, strengthen demand-generation activities, and ensure a consistent availability of trained providers, especially during challenging times like the COVID-19 pandemic.

Despite these challenges, this report offers valuable insights for practitioners, policymakers, and researchers engaged in the field of AYSRH. QI initiatives can be effective mechanisms in boosting the use of contraceptive services. However, for broader application, it is vital to consider contextual factors and implement tailored adaptations to address the needs and preferences of adolescents and youth.^31^^–^^34^ Several avenues for further exploration could deepen our understanding.

First, to ensure the sustainability and effectiveness of health services tailored to adolescents and youth, it is essential to continue future research and additional work in several key areas. Longitudinal assessments are needed to measure the sustainability of the positive changes observed in transformed SDPs and to examine the long-term impact on service quality and contraceptive use. It is also crucial to research ways to secure sustainable funding for adolescents and youth health services, including exploring innovative and sustainable financing models. Institutionalizing youth leadership within health systems requires special attention, especially to encourage their engagement in the planning, implementation, and evaluation of health services that concern them. Maintaining political will at various government levels is essential to ensure ongoing support for adolescents and youth health initiatives and ensure that policymakers maintain their commitment. Finally, it is important to further refine the information system for AYSRH, improving data collection, analysis, and use of data. These research and development efforts require a holistic approach that includes considering the sociocultural and economic determinants influencing the use of health services by adolescents and youth.

Second, to maintain the gains achieved, the institutionalization of QA/QI initiatives within the local government systems is crucial. This involves integrating QA tools and practices into the supportive supervision systems of the local governments and continuing to invest in QI strategies to increase the use of SRH services among adolescents and youth. A proactive response to internal or external factors that may affect service quality is necessary to ensure the strong continuity of services in SDPs.

Third, integrating the WHO building blocks framework is essential for strengthening health systems and supporting sustainability of quality services. To support the sustainable expansion of AYFHS, the focus needs to be on the development of health infrastructure, demand generation in communities, continuous training of health service providers, availability of contraceptive methods, and collaboration between local governments, communities, and international organizations for resource mobilization, advocacy for favorable policies, and sharing of best practices. These areas of focus will also support the health system’s resilience to challenges such as economic recessions or pandemics.

Our findings reinforce the importance of investing in QI strategies to maximize the use of SRH services among adolescents and youth. They also underscore the need for a contextual and nuanced approach to ensure enduring results.

In conclusion, the difference in mean contraceptive uptake between poor-quality and good-quality SDPs has interesting implications on how QI could improve contraceptive uptake among adolescents and youth while also supporting improved quality of service delivery. In this nuanced interplay of service utilization data and quality service delivery, our findings have the potential for use in decisions on funding QI initiatives and informing adolescents and youth policy formulation.

## Notes

**Peer Reviewed**

**First Published Online:** April 15, 2024.
